# Longitudinal Health-Related Values Discussions for Patients Undergoing Hematopoietic Stem Cell Transplant: Do Values Change Over Time?

**DOI:** 10.21203/rs.3.rs-9214963/v1

**Published:** 2026-05-12

**Authors:** Jessica I. Goldberg, Abigail G. Cohen, Jaime L. Gilliland, Kristine Naputo, Danielle Romano, Andrew S. Epstein, Judith E. Nelson, William E. Rosa

**Affiliations:** Memorial Sloan Kettering Cancer Center; Memorial Sloan Kettering Cancer Center; Memorial Sloan Kettering Cancer Center; Memorial Sloan Kettering Cancer Center; Memorial Sloan Kettering Cancer Center; Memorial Sloan Kettering Cancer Center; Memorial Sloan Kettering Cancer Center; Memorial Sloan Kettering Cancer Center

**Keywords:** health-related values, hematopoietic stem cell transplant, hematologic malignancy, palliative care, person-centered communication

## Abstract

**Purpose:**

Hematopoietic stem cell transplant (HSCT) for patients with hematologic malignancies (HM) is associated with significant morbidity. Patients who undergo HSCT benefit from receiving palliative care that addresses their health-related values (HRV). We previously reported results of a pilot study incorporating repeated HRV discussions for patients undergoing HSCT and now aim to explore how patients’ values may change throughout the transplant course.

**Methods:**

Consecutive patients with HM receiving HSCT were approached for enrollment. Patients’ HRV (e.g. personhood, sources of strength, concerns about the future) were assessed using a structured guide and repeated at clinically significant milestones during the transplant course (pre-transplant, Day 10–14, Day 30, Day 100, 6 months, and 1-year). Values discussions were analyzed using qualitative content analysis and transitions (changes in value hierarchy) were identified for individual patients and across treatment subgroups.

**Results:**

31 patients enrolled in the study and participated in 149 discussions. There was little change in personhood. Patients expressed the most changes in their values early in the transplant course (day 10–14 and 30); sources of strength and their meaning of living well were the most variable.

**Conclusion:**

Patients with HM undergoing HSCT experience some transitions in their HRV during the first year after transplant. This study suggests that patients may experience changes in the ways that they understand, prioritize, and reflect on their values in response to a HM diagnosis and its treatment. Person-centered communication should be an iterative process to allow for alignment of decision-making with patients’ values.

## Introduction

Hematopoietic stem cell transplantation (HSCT) is a potentially curative or life-extending treatment for patients diagnosed with hematologic malignancies (HM). HSCT also exposes patients to substantial morbidity and risk of mortality over a prolonged period. As patients enter the transplant process and continue facing its challenges and consequences during weeks, months, or even years, they benefit from communication with clinicians that is person-centered – *i.e.*, addressing health-related values (HRV; what matters most in life, facing illness) and timely. However, person-centered communication (PCC) that prioritizes the assessment and incorporation of patients’ HRV is not routinely integrated into transplant care^1^.

**Figure 1:**
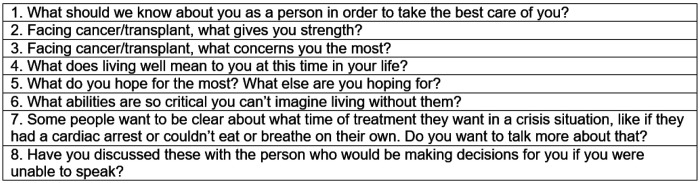
Health-Related Values Discussion Guide

Building on prior work^2,3^, we have developed a structured discussion guide with eight open-ended questions that can be used to assess patients’ HRV (Fig. 1). We previously reported on a pilot study introducing early PCC for patients undergoing HSCT, which found that discussions about HRV were feasible and acceptable for patients and for the transplant team pre-transplant and at multiple time points post-transplant^4^. Given that life events, such as a cancer diagnosis, can prompt changes in the ways that patients’ conceptualize, prioritize, and act upon their values^5–7^, and that the post-transplant clinical course may be complicated, the optimal timing and frequency of values discussions is important. Randomized trial data by our group in patients with solid tumors suggest that advance care planning that entails discussions of HRVs, in addition to video decision aid information about end-of-life care, is associated with more change in patient HRVs^8^. The purpose of the present analysis was to better understand patterns of change in patients’ HRV during the first year after HSCT for HM.

## Methods

The methods of our parent study have been described elsewhere^4^. In short, all consecutive English-speaking patients receiving HSCT (autologous or allogeneic) in two Bone Marrow Transplant (BMT) physicians’ clinics at our comprehensive cancer center were eligible for enrollment. This study was reviewed by the Institutional Review Board at Memorial Sloan Kettering, and as a quality improvement project with no more than minimal risk to participants, was deemed to not require informed consent.

### Health-Related Values Discussions

Patients’ HRV were assessed using a brief, structured guide with eight open-ended questions (Fig. 1)^2,3^. HRV discussions were conducted by a member of the transplant team (physician, nurse practitioner, or registered nurse) based upon the patient’s preference and the standard workflow in the BMT clinic (in-person, telephone, or video call). Discussions were conducted at 6 time points: pre-transplant, peri-transplant (Day 10–14), Day 30, Day 100, 6 months, and 1-year post-transplant. These clinically meaningful points have been used in previous studies involving a similar population^9–11^. Patients’ responses were transcribed verbatim and reviewed with them to assure accuracy before saving in a templated note in the electronic health record. At each time point, the patient was given the option to either update their previous responses as needed or respond anew to the HRV questions.

### Coding

HRV responses were imported into NVivo 14 (Lumivero, Denver, CO) qualitative data analysis software to facilitate data organization and coding. An interdisciplinary team of four coders (JIG, AGC, KN, DR) were trained and advised by the qualitative methods specialist (QMS: JLG). Coders brought expertise in palliative care, social work, and transplant care. Responses to each HRV question (personhood, sources of strength, concerns, living well, critical abilities, hopes, care preferences) were coded separately. Using qualitative content analysis^12,13^, the coders: 1) familiarized themselves with the HRV responses, 2) generated an initial coding framework with general categories, 3) applied the codebook to all responses using an independent and collaborative process, and 4) consolidated the categories into themes. The coders and QMS met to discuss and resolve any coding discrepancies.

### Analysis

Matrices were created to visualize the distribution of the themes and codes at each time point. The most frequently occurring themes were identified and organized into a hierarchy (e.g. 1st, 2nd, 3rd ). Transitions were defined as a change in the hierarchy between two time points. Since patient selection, diseases treated, transplant course, side effect profile and intensity, and expected outcomes of allogeneic and autologous HSCT are different^14,15^, separated matrices was created based on transplant type. Additionally, a matrix was developed for each patient. These matrices allowed the researchers to identify changes by group and by individual.

## Results

Characteristics of the study participants have been presented elsewhere^4^, but in brief, 31 patients were enrolled between March 2021 and March 2022. The majority were male (n=20, 65%), White (n=22, 71%), had a primary diagnosis of multiple myeloma (n=15, 48%), and received an autologous HSCT (n=18, 58%) (Table 1).

### Health-Related Values Discussions

In total, there were 149 HRV discussions (pre-HSCT=29, Day 10-14=28, Day 30=25, Day 100=23, 6-month post-HSCT=22, 1-year post-HSCT=22) and over seventy percent of patients (n=22) participated in an HRV discussion at each of the six time points. Two patients did not participate in any of the discussions; one was deemed by the transplant team to be experiencing multiple acute medical issues, and one required an urgent HSCT. Patients who did not complete the initial discussion pre-transplant were not approached at follow-up time points. The discussions took less than twenty minutes to complete, and most were conducted by a nursing professional (n=135, 91%) via telephone (n=79, 53%) (Table 2).

### Values Themes

113 total codes were identified and organized into 29 themes (Table 3). In both subgroups, the peri-transplant (day 10-14) and day 30 time points represented the times when the most patients had changes in their HRV (sources of strength, concerns, living well, hopes, critical abilities, care preferences). Responses regarding sources of strength and qualities of living well were the most variable over time. There was little reported change in patients’ personhood responses, regardless of the treatment subgroup or time point (Table 4).

#### Personhood (“What should we know about you as a person in order to take the best care of you?”)

In pre-transplant responses, patients together identified a variety of attributes defining their personhood. Many emphasized their willingness to comply with the transplant regimen, while continuing to seek information and voicing their concerns.

“It’s important for me to know every detail of my care, whether good or bad. I don’t like to complain but I will speak up if I am in pain or have questions about my care. I am a rule follower and follow directions by the book.” Patient 17, autologous HSCT, pre-transplant

Patients also described the importance of setting boundaries (e.g. with family and medical providers) and sources of meaning or fulfillment (e.g. role as a parent, participation in sports). At later time points, several patients discussed how their cancer diagnosis was a challenge to their worldview, even months after the transplant.

“I’ve always been a positive person. Prior to all of this nothing ever stopped me or brought me down. I’m so tired of being negative and thinking the worst all the time. I don’t like to give in and I’m not that kind of person.” Patient 20, allogeneic HSCT, 6 months post-transplant

#### Sources of Strength (“Facing cancer/transplant, what gives you strength?”)

Before transplant, patients identified their family and friends, and themselves as a primary source of strength.

“I can always overcome obstacles- I can do this. I’ve always had self-confidence. I can overcome this.” Patient 9, allogeneic HSCT, pre-transplant

At the peri- and post-transplant time points, patients in the autologous subgroup primarily described gaining strength from internal sources (e.g. faith or religion, or sense of hope). In comparison, patients in the allogeneic group highlighted an external source of strength, trust in the medical team, early (Day 10-14) and at multiple later time points. (Day 30, 6 months post-transplant).

“I feel supported by my doctors and nurses and am very appreciative of that.” Patient 19, allogeneic HSCT, Day 10-14

Several patients experienced a transition in their source of strength away from an external source (e.g. family and friends) and towards an internal source (e.g. faith) in the days immediately after transplant. For example, patient 9 (autologous HSCT) identified their *“family and friends”* pre-transplant, and at the peri-transplant time point, described seeking strength from their *“faith in God… I pray every night”.*

#### Concerns (“Facing cancer/transplant, what concerns you the most?”)

At the pre-transplant time point, patients acknowledged many concerns regarding quality of life, survival or death, physical symptoms, psychological symptoms, loss, and family. Patients described concerns about the loss of normalcy after the cancer diagnosis.

“I guess my biggest thing is not existing as I know myself… Before all this I was vital, hopeful, optimistic. I’ve lost quite a bit of that. I’ve always been so active and strong. I worry about not being back to myself.” Patient 20 allogeneic HSCT, pre-transplant

Concerns about loss, survival, and death were frequently discussed in both subgroups at all time points.

“My biggest fear is telling [my wife] I’m going for a check-up and not coming home… I feel like I’m living on borrowed time.” Patient 29, allogeneic HSCT, Day 100

Shortly after the transplant, patients frequently had transitions in their concerns for the future. Patients described new worries about physical symptoms. One patient (patient 21, autologous HSCT) reported pre-transplant that their biggest concern was *“not being about to be there for [my] family in the way that they need [me] to be”*, but by day 10-14, described a worry that *“my kidney could fail… bleeding and mouth sores”.*

#### Living Well (“What does living well mean to you at this time in your life?”)

Patients described characteristics of living well, including ensuring their emotional well-being, and a return to their prior self, profession, or level of independence. Many patients identified that participating in activities that bring joy or meaning and having a connection with others were markers that they were living well.

“Being with family and having friends over. My daughter and grandsons are coming to visit for two weeks.” Patient 4, autologous HSCT, Day 60

The patients in the autologous subgroup described the importance of participating in physical activities at each time point in the transplant course. In contrast, it was reported only on day 30 and 100 by patients in the allogenic subgroup.

“…the ability to remain active such as hiking, skiing, swimming, and cycling. Being able to hopefully… see the world.” Patient 14, allogeneic HSCT, Day 30

Several patients reported experiencing transitions in how they defined living well, which corresponded to changes in their physical functioning. For example, patient 30 (autologous HSCT) described living well pre-transplant as, *“staying in shape, being able to do the things I used to be able to do and see family”.* However, 6-months after the transplant, living well for this patient meant *“… getting used to my new normal which has physical limitations. So, living well now means eating ice cream and cream, sleeping better…”.*

#### Hopes (“What do you hope for the most?”)

The patients reported a diverse list of hopes, such as survival or a cure, a good end of life, physical comfort, family functioning, emotional well-being or adjustment, and a shift in their desire to find meaning. The most frequently reported hopes, regardless of time point or treatment subgroup, were for a return to normalcy, and for a chance at cure or survival.

“I know a cure is unrealistic, but I hope to be in a remission to the point where I can go about my normal activities without worry.” Patient 3, autologous HSCT, Day 10-14

#### Critical Abilities (“What abilities are so critical you can’t imagine living without them?”)

Patients identified critical abilities that they could not imagine living without, including a sense of social connectedness or belonging, intellectual abilities, and emotional or spiritual activities. The ability to participate in physical activities was the most frequently reported critical ability and was mentioned at every time point, in both treatment subgroups.

“…walking without a walking stick. I hope I can get on the dance floor as soon as possible.” Patient 28, autologous HSCT, pre-transplant

Multiple patients had transitions in what they considered a critical ability as they advanced through the transplant course. One such patient (patient 16, allogeneic HSCT) explained before transplant that *“the whole mobility thing”* was a critical ability they couldn’t imagine living without. However, at day 30, this patient explained, *“…the biggest one to me would be losing my cognitive ability. I’ve come to terms with the whole physical abilities. I’ve had to adjust my expectations. But cognitive ability is the most important”.*

#### Care Preferences (“Some people want to be clear about what type of treatment they want in a crisis situation, like if they had a cardiac arrest or couldn’t eat or breathe on their own. Do you want to talk more about that?”)

Patients’ responses regarding end-of-life preferences were varied, covering the spectrum from avoidance of life-prolonging therapy in the context of uncontrollable disease to full life-supporting measures regardless of prognosis.

“I have a Five Wishes document and will bring it to the hospital. I uploaded the document in the portal. I want this to define and articulate my preferences.” Patient 11, allogeneic HSCT, Day 10-14

The specific end-of-life preferences were largely consistent over time, from the pre-transplant period to 1-year after transplant. Several patients initially did not want to discuss their end-of-life preferences but did experience a transition in both their desire to discuss their wishes and their interest in speaking with others about their preferences. For example, patient 31 (allogeneic HSCT) declined to discuss these care preferences before transplant or at day 10-14. However, by day 30 this patient now clarified that they *“would want CPR and ventilation. The longer the life the better and I would want to be left alive until I am taken away by a higher power”.* On day 30, the patient reported that they had not spoken about their wishes with anyone in their life, and by day 100 they had discussed their preferences with their wife and friends.

## Discussion

This qualitative study examined HRV of patients undergoing HSCT and evaluated if values changed during the long and often difficult transplant process. While there is evidence that end-of-life preferences may change over time, core values are generally expected to be more enduring^5^. However, our data suggests that at different time points, beginning at the pre-transplant evaluation and continuing through one year after transplant, certain aspects of patients’ values may evolve. Most of these transitions seemed to occur in the days and weeks shortly after transplant (day 10–14 and day 30), which corresponds to the time when patients experience the most significant symptom burden. Yet we also found transitions in themes (change in hierarchy) of patient-reported values throughout the first year after transplant, affirming that person-centered communication cannot be static but rather should be a dynamic and iterative process as a basis for medical decision-making in accordance with patients’ core values.

Some past studies suggest that values will remain stable because they are intrinsic to a person’s identity, although their priority or hierarchy may change based on disease status^16,17^. Like identity, personhood relates to what makes an individual unique. In our sample, we found that patients’ descriptions of their personhood did tend to be consistent over time, but their other values, less tied to their intrinsic identity, were more likely to evolve.

Exploration of patients’ personhood and values^20^ was not an explicit component of palliative care interventions that were shown to benefit patients with HM^11,21^, although it is an essential part of palliative care, which is grounded in person-centered care. As a result, there remain gaps between guideline recommendations for the integration of palliative care into routine hematologic oncology^22,23^ and actual practice of clinicians caring for patients with HM^24,25^. Data from this study suggests the benefit of an individualized, iterative approach to discussion of patient values, structured by specific questions inviting open-ended responses, with an exploration of personhood at a baseline time point, and a revisiting of certain values domains (e.g. sources of strength, qualities of living well) at subsequent intervals. Since repeated discussions may have burdens as well as benefits for patients and clinicians, our ongoing research seeks to identify more clearly whether certain time points are more meaningful than others in mapping the trajectory of values over time, and whether clinical events (e.g., worsening symptoms, treatment change, disease recurrence) might also be important as prompts for revisiting values.

Although some clinicians worry that patients – particularly those undergoing potentially curative treatments – will be inclined to avoid discussions about end-of-life preferences, most HSCT patients in this cohort did articulate such preferences, even with the option to defer the discussion. In addition, they were willing to revisit these preferences over time, and their preferences were largely stable across different time points. This is in contrast to other studies where patients were reluctant to revisit discussions about end of life care beyond the pre-transplant period^18,19^. At the pre-transplant evaluation with patients in our study, the clinician sought explicitly to normalize the discussion of values and preferences as part of the ongoing transplant care, to be revisited at specific intervals without regard to the patient’s clinical course after transplant. By decoupling the discussion from prognosis and providing patients with a positive communication experience early on, this approach may have decreased patients’ apprehension and increased acceptability and comfort for clinicians about discussions at subsequent time points.

This study has several limitations which impact its generalizability. The patients were enrolled from a single institution, and although there was some heterogeneity in the demographic and clinical characteristics, the sample size was small and drawn from only 2 transplant clinics. However, our sample included patients who had autologous and allogeneic transplants in order to capture potential differences based on the type of transplant. In this institution, the results of our pilot study provided the basis for generalizing the values discussion intervention across all transplant and cellular therapy clinics. There was a drop-off in the completion of the HRV discussions at later time points, making it more difficult to observe transitions across all six discussions for all patients. Still, over seventy percent of patients participated in an HRV discussion at each of the six time points. We did not have sufficient statistical power to determine changes over time quantitatively, but instead we examined trends using qualitative analysis methods.

In summary, we found that patients with HM undergoing HSCT were receptive to participating in serial discussions of HRV, and that some aspects of HRV, especially those less connected to ones personhood, may evolve during the first year post transplant. The patients in this study were able to articulate their values in an iterative process as part of routine oncologic care, decoupled from prognosis. Further studies will add to our understanding about the optimal ways to integrate person-centered palliative care for patients with HM in a manner that is proactive, and responsive to individual’s needs as they evolve over time and in response to clinical events.

## Figures and Tables

**Table 1.** Demographic and Clinical Characteristics of Participants (N=31)

Variable	Median (Range)	N (%)

Age (years)	66 (60-69)	

Gender		
	Male	20 (65)
	Female	11 (35)

Race		
	White	22 (71)
	Black	4 (13)
	Asian	5 (!6)

Primary disease		
	Multiple Myeloma	15 (48)
	AML	9 (29)
	Other	7 (23)

HSCT type		
	Autologous	18 (58)
	Allogeneic	13 (42)

Note. N= number, Other= myeloproliferative neoplasm, myelodysplastic syndrome, amyloid, HSCT= hematopoietic stem cell transplant; Adapted from Cohen, 2025^4^

**Table 2.** Characteristics of Health-Related Values Discussions

Variable	Median (Range)	N (%)

Completed discussions (N=31 patients)		
Pre-HSCT		29 (94)
Peri-HSCT (Day 10-14)		28 (90)
Day 30		25 (81)
Day 100		23 (74)
6 months post-HSCT		23 (74)
1 year post-HSCT		22 (71)

Discussion length in minutes (N=149 discussions)		
Pre-HSCT	17.7 (3-35)	
Subsequent time points	13.3 (1-50)	

Clinician conducting discussion (N=149 discussions)		
Nurse		57 (38)
Nurse practitioner		78 (52)
Physician		14 (9)

Communication method (N=149 discussions)		
In-person		55 (37)
Telephone		79 (53)
Video call		14 (9)
Hybrid (in-person + telephone)		1 (1)

Note. N= number, HSCT= hematopoietic stem cell transplant; Adapted from Cohen, 2025^4^

**Table 3.** Summary of Themes and Codes

HRV Question	Themes	Codes

Personhood	a)Aspects of character	Stubborn Resilient Compliant Proactive Independent/strong Direct Easygoing/understanding Defiant Active Desire to please the medical team
	b) Boundary setting	Private Want autonomy
	c) Sources of meaning or fulfillment	Importance of family and friends Being an advocate Being a fighter Productive Altruistic
	d) Life/world view	Optimist Pessimist Realistic

Sources of Strength	a) Internal	Self Hope Faith or religion
	b) External	Science or data Family and friends Faith or religion Trust and belief in care team Activities that bring enjoyment Financial stability

Concerns	a) QOL concerns	Change in health or happiness
	b) Survival or death	Dying Relapse Treatment failure or complications
	c) Physical symptoms	Feeling worse Nausea Pain
	d) Loss	Loss of quality of life Uncertainty Loss of normalcy Being disabled Loss of independence or freedom
	e) Family	Being a burden on loved ones Wellbeing of family

Living Well	a) Activities that bring joy or meaning	Travel Spending quality time with family and friends Reading Continuing to learn Praying Participating in important life events of loved ones Art
	b) Connection with others	Spending quality time with family and friends Participating in important life events of loved ones Helping others
	c) Physical activities	Being healthy Being active Travel Being independent Being physically comfortable
	d) Emotional wellbeing	Not worrying Being independent Praying Reading Helping others Returning to normalcy
	e) Professional role, independence, return to prior self	Retiring or working less Working

Hopes	a) Cure or survival	Longevity Cure or treatment success Recovery or survival
	b) End of life	Peaceful death
	c) Physical comfort	Free from treatment complications Free from limitations from disease Free from pain Being more active Health Quality of life Strength
	d) Family functioning	Spend time with family Conflict resolution with family Wellbeing of family
	e) Normalcy	Returning to work Being at home or out of hospital Travel Being more active
	f) Emotional well-being or emotional adjustment	Free from worry or uncertainty Positivity Acceptance or adjustment to new normal
	g) Priority or goal shift, finding meaning	Health Travel Retirement Help people Being more active

Critical Abilities	a) Social connectedness, belonging, relationships	Being with family or friends Loving Taking care of family
	b) Physical abilities	Working Being active Being independent Hearing or sight Making art
	c) Intellectual abilities	Cognition
	d) Emotional or spiritual abilities	Loving Having fun Making art Adaptability Maintaining a positive attitude

Care Preferences	a) Discussion about care preferences	Desire not to discuss Desire to discuss further Defer decisions to health care agent
	b) EOL preferences	Full life support measures Resuscitation if chance of recovery Not to pursue aggressive therapy if disease is uncontrollable Not to be on a ventilator for a prolonged time No dialysis

Note. QOL= quality of life, EOL= end of life

**Table 4.** Thematic Changes Over Time by HSCT Subgroup

HRV Question	HSCT Subgroup	Pre-HSCT	Changes at Day 10-14	Changes at Day 30	Changes at Day 100	Changes at 6 months post-HSCT	Changes at **1 year post-HSCT**

**Personhood**	Auto	1. Aspects of character 2. Sources of meaning and fulfillment	N/A	N/A	N/A	N/A	N/A
	
	Allo	1. Aspects of character 2. Sources of meaning and fulfillment	N/A	(−) Sources of meaning and fulfillment	N/A	(+) Boundary setting	N/A

**Sources of Strength**	Auto	1. Friends and family 2. Self	(+) Faith and religion (−) Self	(+) Hope	N/A	(−) Hope	N/A
	
	Allo	1. Friends and family 2. Self	(+) Trust in care team	(+) Coping	(−) Family and friends	(+) Hope	(−) Trust in care team

**Concerns**	Auto	1. Loss 2. Survival or death	(+) Physical symptoms	N/A	(−) Physical symptoms	N/A	(+) QOL concerns
	
	Allo	1. Loss 2. Survival or death	(+) Physical symptoms	N/A	N/A	N/A	(−) Physical symptoms

**Living Well**	Auto	1. Activities that bring joy or meaning 2. Physical activities	(+) Connection with others	(+) Professional role, independence, return to prior self (−) Connection with others	N/A	(−) Professional role, independence, return to prior self	(+) Professional role, independence, return to prior self
	
	Allo	1. Professional role, independence, return to prior self 2. Connection with others	(+) Activities that bring joy or meaning (−) Connection with others	(+) Connection with others (+) Physical abilities (−) Activities that bring joy or meaning	(−) Professional role, independence, return to prior self	(+) Activities that bring joy or meaning (−) Physical abilities	N/A

**Hopes**	Auto	1. Normalcy 2. Cure or survival 3. Family functioning	N/A	(+) Physical comfort	N/A	N/A	N/A
	
	Allo	1. Cure or survival	(−) Physical comfort	(+) Family functioning	(−) Family functioning	N/A	(−) Cure or survival
		2. Physical comfort 3. Normalcy					

**Critical** **Abilities**	Auto	1. Physical abilities: being active and independent	N/A	N/A	N/A	N/A	N/A
	
	Allo	1. Physical abilities: being active and independent	N/A	(+) Working	N/A	(−) Working	(−) Being independent

**Care** **Preferences**	Auto	1. Resuscitation if chance of recovery	N/A	N/A	(+) Health care agent to decide	(−) Health care agent to decide	(+) Health care agent to decide
		2. Full life support measures					
		3. Discussion about care preferences					
	
	Allo	1. Health care agent to decide 2. Discussion about care preferences	(+) Not to be on the ventilatory for prolonged time	(−) Not to be on the ventilatory for prolonged time	(−) Health care agent to decide	N/A	N/A

Note. HRV=health-related value, HSCT= hematopoietic stem cell transplant, Auto= autologous, Allo=allogeneic, (+)= addition of theme, (−)= removal of theme
